# Synthesis of Terpyridines: Simple Reactions—What Could Possibly Go Wrong?

**DOI:** 10.3390/molecules24091799

**Published:** 2019-05-09

**Authors:** Dalila Rocco, Catherine E. Housecroft, Edwin C. Constable

**Affiliations:** University of Basel, Department of Chemistry, BPR 1096, Mattenstrasse 24a, CH-4058 Basel, Switzerland; dalila.rocco@unibas.ch (D.R.); catherine.housecroft@unibas.ch (C.E.H.)

**Keywords:** terpyridines, 3,2′:6′,3″-terpyridine, cyclohexanol derivative, condensation, heterocyclic

## Abstract

The preparation of 2^4^-functionalized 1^2^,2^2^:2^6^,3^2^-terpyridines (4′-functionalized 3,2:6′,3″-terpyridines) by the reaction of three 4-alkoxybenzaldehydes with 3-acetylpyridine and ammonia was investigated; under identical reaction conditions, two (R =*^n^*C_4_H_9_, C_2_H_5_) gave the expected products whereas a third (R = *^n^*C_3_H_7_) gave only a cyclohexanol derivative derived from the condensation of three molecules of 3-acetylpyridine with two of 4-(*n*-propoxy)benzaldehyde. A comprehensive survey of “unexpected” products from reactions of ArCOCH_3_ derivatives with aromatic aldehydes is presented. Three different types of alternative product are identified.

## 1. Introduction

The 48 isomeric structures obtained by the linking together of three pyridine rings by three single bonds are known collectively as the terpyridines [1]. One of the commonest metal-binding scaffolds encountered in supramolecular and materials chemistry is 2,2′:6′,2″-terpyridine (**1**, 2,2′:6′,2″-tpy) [2,3,4,5,6,7]: Note that in this article, we use the nomenclature that is established in the community for the terpyridines, rather than the preferred IUPAC names (PINs) of 1^2^,2^2^:2^6^,3^2^-terpyridine, 1^3^,2^2^:2^6^,3^3^-terpyridine and 1^4^,2^2^:2^6^,3^4^-terpyridine for 2,2′:6′,2″-terpyridine, 3,2′:6′,3″-terpyridine and 4,2′:6′,4″-terpyridine respectively [8]. Although the high kinetic and thermodynamic stability of 2,2′:6′,2″-tpy complexes confers unique properties, the main reason why this metal-binding domain is so commonly observed is the ease of synthesis of derivatives with a wide variety of substituents, in particular at the 4′-position of the central ring.

**Scheme 1:**
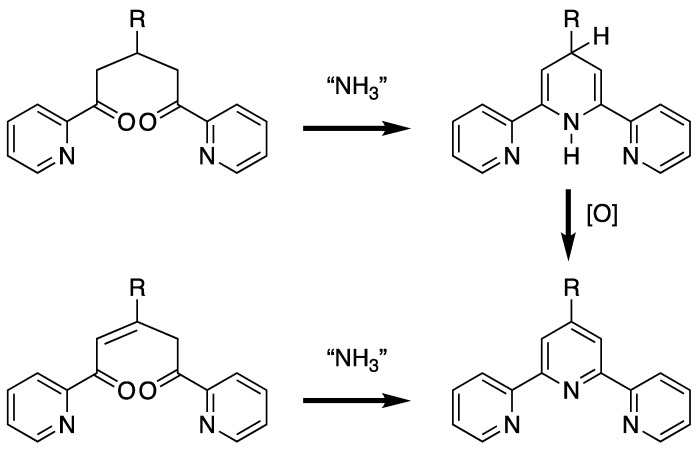
The typical synthetic strategy for a 2,2′:6′,2″-tpy involves the cyclization of 1,5-bis(pyridin-2-yl)pentane-1,5-dione or 1,5-bis(pyridin-2-yl)pent-2-ene-1,5-dione or equivalent with ammonia. In the case of the 1,5-bis(pyridin-2-yl)pentane-1,5-dione, the intermediate dihydro-compound (the 1′,4′-dihydro-2,2′:6′,2″-terpyridine is shown) requires an oxidation to generate the 2,2′:6′,2″-tpy.

Although a vast array of synthetic strategies have been developed for the preparation of 2,2′:6′,2″-tpy derivatives, the majority are based upon the synthesis of an intermediate 1,5-bis(pyridin-2-yl)pentane-1,5-dione or 1,5-bis(pyridin-2-yl)pent-2-ene-1,5-dione (or their synthetic equivalents) which are subsequently cyclized to generate the central pyridine ring by reaction with a source of ammonia. Although the 1,5-bis(pyridin-2-yl)pent-2-ene-1,5-dione is at the correct oxidation state for the formation of the pyridine upon cyclization, the 1,5-bis(pyridin-2-yl)pentane-1,5-dione yields a 1′,2′- or 1′,4′-dihydro-2,2′:6′,2″-terpyridine which formally requires oxidation to the desired 2,2′:6′,2″-tpy (Scheme 1).

**Figure 1:**
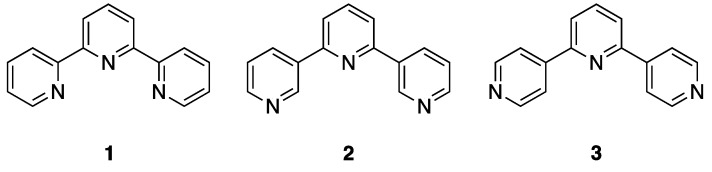
The structure of the most commonly encountered terpyridine, 2,2′:6′,2″-terpyridine (**1**), together with the closely related compounds 3,2′:6′,3″-terpyridine (**2**) and 4,2′:6′,4″-terpyridine (**3**).

**Scheme 2:**
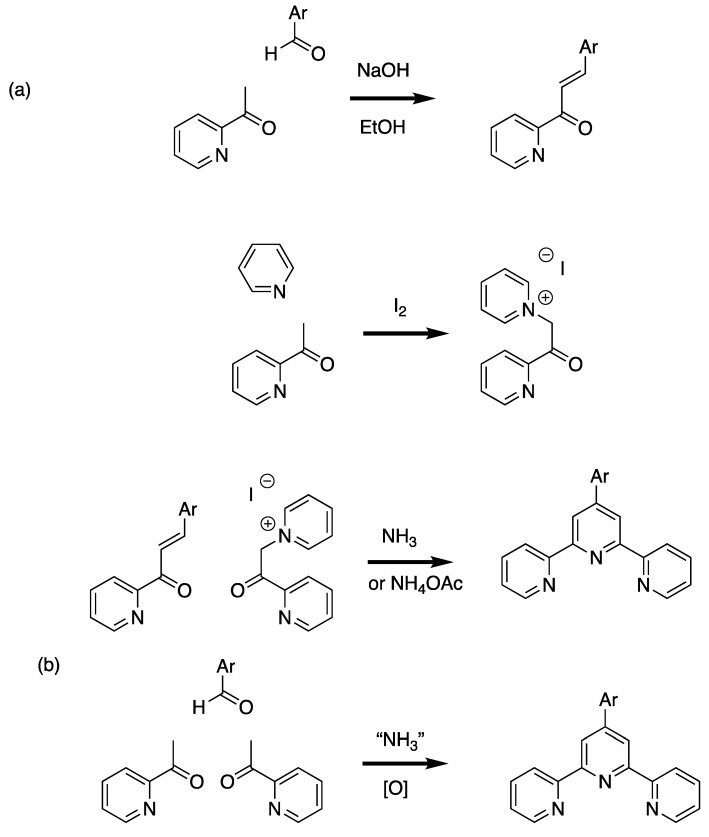
Variations on the Kröhnke reaction for the preparation of 4′-aryl-2,2′:6′,2″-tpy ligands. (**a**) The classical Kröhnke route in which an enone (chalcone) is prepared from the aldol condensation of 2-acetylpyridine with an aromatic aldehyde followed by reaction with a ″2-*pyridacylpyridinium*″ salt (obtained from an Ortoleva-King reaction) in the presence of an ammonia source (often ammonium acetate) to give the 4′-aryl-2,2′:6′,2″-tpy. (**b**) The simpler ″one-pot″ approach in which two equivalents of 2-acetylpyridine and the aromatic aldehyde are reacted directly or sequentially with a source of ammonia. This latter reaction may be in solvent free-conditions by grinding the reactants, use benign solvents such as PEG or conventional solvents. Replacement of the 2-acetylpyridine by 3-acetylpyridine or 4-acetylpyridine allows the synthesis of 4′-aryl-3,2′:6′,3″-tpy and 4′-aryl-4,2′:6′,4″-tpy ligands.

The most common implementations of this strategy are based on the approach of Kröhnke (Scheme 2) [9]. In the classical Kröhnke synthesis, an intermediate enone (with the trivial name *chalcone* when two aromatic substituents are present [10]) obtained from the Claisen-Schmidt condensation [11,12] of 2-acetylpyridine with an aromatic aldehyde is reacted with an ammonia source and a “2-*pyridacylpyridinium*” salt; the latter is conveniently obtained by the Ortoleva-King reaction of 2-acetylpyridine with pyridine and iodine [13,14,15]. A convenient alternative is the direct reaction of two equivalents of 2-acetylpyridine with the aromatic aldehyde to give the 4-aryl-1,5-bis(pyridin-2-yl)pentane-1,5-dione directly or sequentially via the chalcone. Particularly attractive variations are solvent-free (or benign solvent) reactions and one-pot syntheses in which the reactants and an ammonia source, often ammonia and/or ammonium acetate are reacted directly or sequentially with the aromatic aldehyde and an acetylpyridine [16,17,18,19,20]. By replacing the 2-acetylpyridine by 3-acetylpyridine or 4-acetylpyridine, the synthetic approaches are readily adapted to the preparation of the symmetrical terpyridine isomers 3,2′:6′,3″-terpyridine (**2**, 3,2′:6′,3″-tpy, 1^3^,2^2^:2^6^,3^3^-terpyridine) and 4,2′:6′,4″-terpyridine (**3**, 4,2′:6′,4″-tpy, 1^4^,2^2^:2^6^,3^4^-terpyridine) (Figure 1). Although 2,2′:6′,2″-tpy is the most common motif encountered in coordination chemistry, 3,2′:6′,3″-tpy and 4,2′:6′,4″-tpy are increasingly finding application in the metal-directed formation of coordination networks [21,22,23,24]. In coordination networks incorporating 3,2′:6′,3″-tpy and 4,2′:6′,4″-tpy, the nitrogen atom of the central pyridine ring (ring 2 in the IUPAC recommendation), is never coordinated to a metal ion.

**Figure 2:**
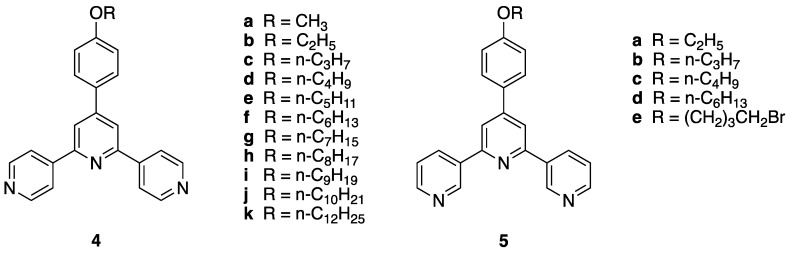
Structures of 4′-(4-alkyloxyphenyl)-4,2′:6′,4″-terpyridines and 4′-(4-alkyloxyphenyl)-3,2′:6′,3″-terpyridines. Compounds **5a**–**c** are the subject of this publication.

In this paper, we discuss the synthesis of a series of 3,2′:6′,3″-tpy ligands (Figure 2) and describe the formation of an unexpected product in one case. The paper also provides a comprehensive review of alternative products which have been obtained from syntheses of terpyridines using the synthetic approaches above. We take this opportunity to issue a *caveat*: when utilizing these synthetic methods, the terpyridine products should be purified and fully characterized.

## 2. Results and Discussion

### 2.1. Strategy and Ligand Design

We have shown that in the 4,2′:6′,4″-tpy compounds **4a**–**k** (R = *n*-C_n_H_2n+1_, *n* = 1–10, 12, Figure 2), the length of the alkyl chain on the alkyloxy substituent can influence the packing and the topology of coordination networks generated upon reaction with metal salts [22,25,26,27,28,29,30]. We are now beginning a systematic investigation of the effect of substituents on the assembly of coordination networks based on 3,2′:6′,3″-tpy ligands and commenced with the synthesis of derivatives **5a**–**c**. Compound **5d** has previously been reported [31]. The synthetic approach was a standard one-pot synthesis, involving the reaction of 3-acetylpyridine with the appropriate 4-alkyloxybenzaldehyde in basic solution, followed by the addition of aqueous ammonia.

### 2.2. Results

#### 2.2.1. 4′-(4-Ethoxyphenyl)-3,2′:6′,3″-terpyridine and 4′-(4-butoxyphenyl)-3,2′:6′,3″-terpyridine

**Figure 3:**
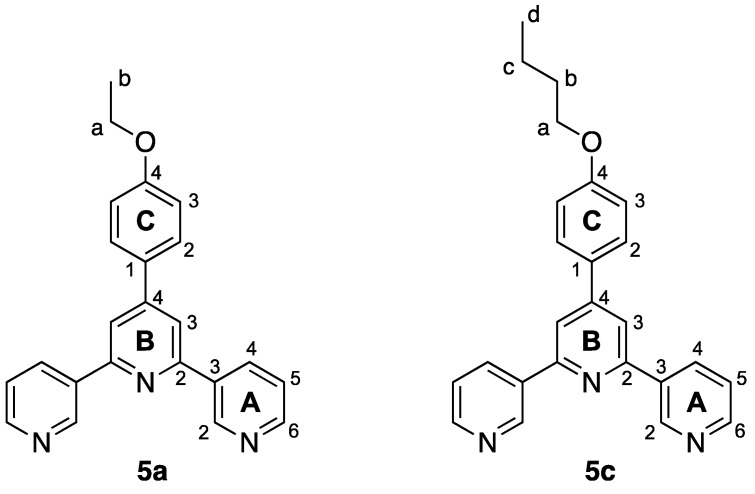
Structures of compounds **5a** and **5c** with atom labelling for NMR spectroscopic assignments.

4′-(4-Ethoxyphenyl)-3,2′:6′,3″-terpyridine (**5a**) and 4′-(4-butoxyphenyl)-3,2′:6′,3″-terpyridine (**5c**) (Figure 3) were prepared following the one-pot method of Wang and Hanan [18]. 4-Ethoxybenzaldehyde or 4-butoxybenzaldehyde were reacted with 3-acetylpyridine in EtOH in the presence of KOH. After addition of aqueous NH_3_, **5a** and **5c** precipitated over a period of about 16 h as white solids in 16.7 and 31.1% yields, respectively.

**Figure 4:**
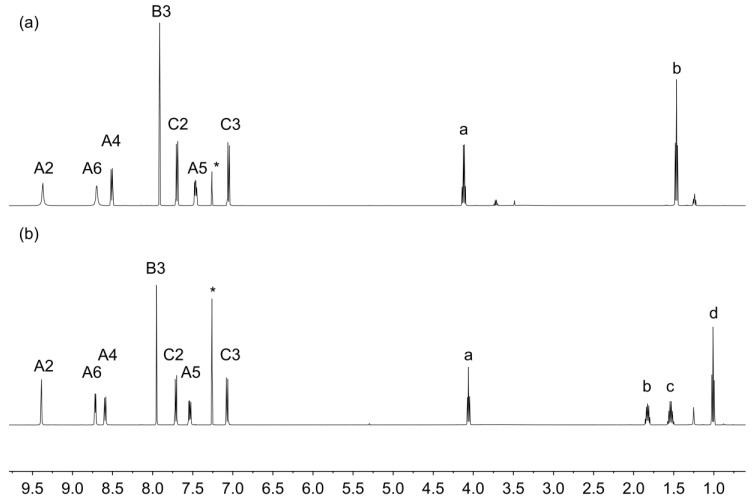
^1^H NMR spectra (500 MHz, CDCl_3_, 298 K) of (**a**) **5a** and (**b**) **5c**. See Figure 3 for the atom labelling scheme. * = residual CHCl_3_.

**Figure 5:**
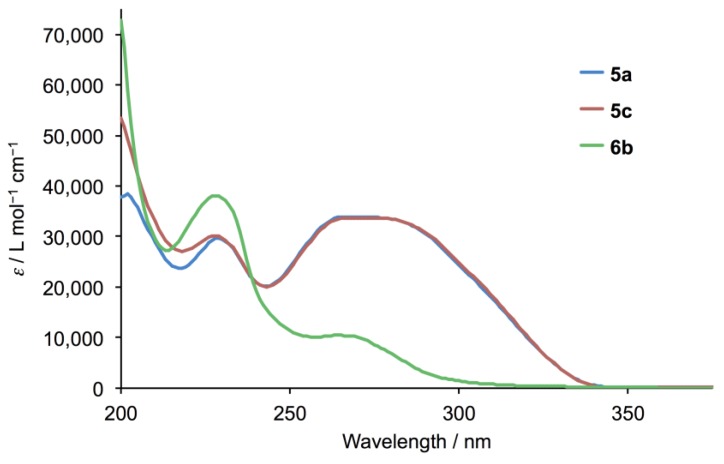
Solution absorption spectra of **5a**, **5c** and **6b** (MeCN, 3.3 × 10^–5^ mol dm^–3^).

The electrospray (ESI) mass spectra of compounds **5a** and **5c** showed base peaks at *m*/*z* 354.08 and 382.16, respectively, arising from the [M + H]^+^ ions (Figures S1 and S2, see Supporting Information). The solution ^1^H and ^13^C NMR spectra were consistent with the structures shown in Figure 3 and were assigned using COSY, NOESY, HMQC and HMBC methods. Figure 4 displays a comparison of the ^1^H NMR spectra, and confirms that the introduction of the different alkyloxy substituents has no significant influence on the spectroscopic signature of the 4′-phenyl-3,2′:6′,3″-tpy unit. ^13^C{^1^H} NMR spectra are compared in Figure S3. As expected, the solid-state IR spectra of **5a** and **5c** are very similar (Figures S4 and S5) and the solution absorption spectra (Figure 5) show intense absorptions in the UV region arising from spin-allowed π*←π and π*←*n* transitions.

#### 2.2.2. A Reaction that Works with Ethoxy and Butoxy Homologues, Fails for 4′-(4-propoxyphenyl)-3,2′:6′,3″-terpyridine

**Figure 6:**
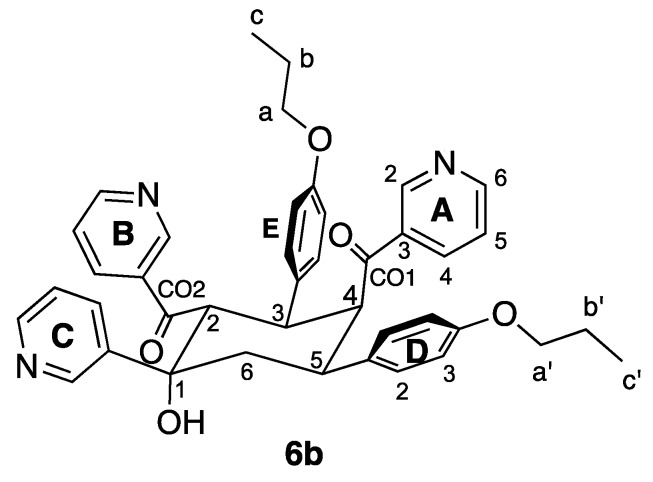
Structure of compound **6b** with atom labelling for NMR spectroscopic assignments.

During attempts to prepare 4′-(4-propoxyphenyl)-3,2′:6′,3″-terpyridine (**5b**, Figure 2) by the reaction of 4-propoxybenzaldehyde with 3-acetylpyridine in the presence of KOH in ethanol, under identical conditions to those for the successful preparation of ligands **5a** and **5c**, followed by addition of aqueous NH_3_, we noted that precipitation of a product began before ammonia was added. The white solid that was isolated exhibited IR (Figure S6) and absorption spectra (Figure 5) with different profiles from those of **5a** and **5c**. In particular, the IR spectrum of the product exhibited a sharp absorption at 3495 cm^–1^ attributable to an OH group. These observations were unexpected since we have previously reported the successful synthesis of the analogous series of 4,2′:6′,4″-tpy derivatives **4a**–**4k** (Figure 2) by the Hanan one-pot strategy [25]. The ESI mass spectrum of the product showed a base peak at *m/z* 656.31 (Figure S7) suggesting the formation of the cyclic product **6** shown in Figure 6.

**Figure 7:**
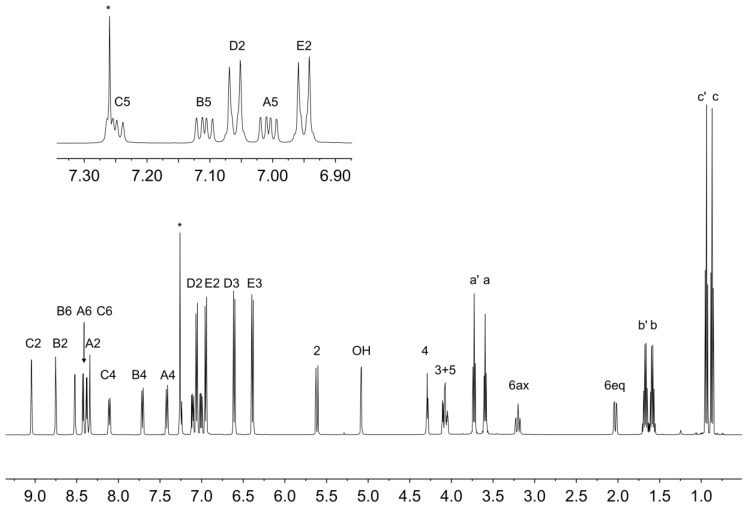
^1^H NMR spectrum (500 MHz, CDCl_3_, 298 K) of compound **6b**. See Figure 6 for atom labelling. * = residual CHCl_3_.

The solution ^1^H NMR spectrum of **6b** is shown in Figure 7 and is consistent with the presence of three pyridine environments (rings A, B and C) and two 4-propoxyphenyl environments (rings D and E). The spectrum was assigned using COSY, NOESY (Figure S8), HMQC (Figure S9) and HMBC (Figure S10) methods with critical NOESY crosspeaks being between H^OH^/H^C2^, H^D3^/H^a′^, H^E3^/H^a^, H^D2^/H^5^, H^D2^/H^6ax^, H^E2^/H^3^ and H^E2^/H^2^.

**Figure 8:**
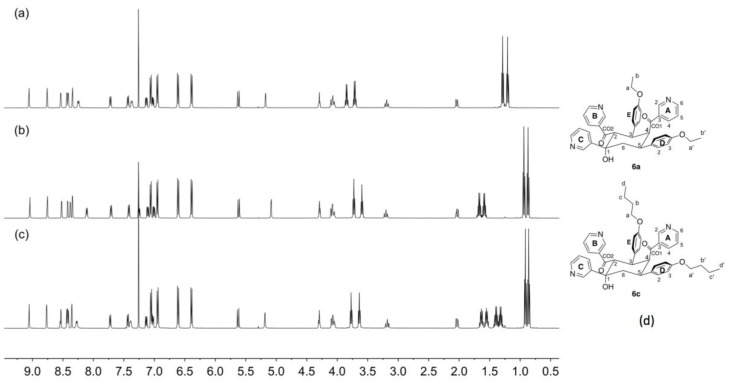
A comparison of the ^1^H NMR (500 MHz, CDCl_3_, 298 K) spectra of the cyclic products (**a**) **6a**, (**b**) **6b** and (**c**) **6c**. The singlet at *δ* 7.26 ppm in each spectrum is residual CHCl_3_. (**d**) The structures of **6a** and **6c** with atom labelling for NMR spectroscopic assignments (see Section 4.5 and Section 4.6).

The spectroscopic characterization confirms the formation of **6b** although it offers no indication as to why this product precipitates from solution only in the case of the propoxy substituent. We noted that the reaction only involves the aldehyde and the 3-acetylpyridine and were prompted to perform the reactions in the absence of ammonia. We repeated the reaction of 4-propoxybenzaldehyde with 3-acetylpyridine at a 1:2 molar ratio in ethanol with KOH but without the addition of NH_3_. This led to the formation of **6b** (confirmed by NMR spectroscopy, Figure 8b) in 42.0% yield. We then performed the analogous reactions of 4-ethoxybenzaldehyde or 4-butoxybenzaldehyde with 3-acetylpyridine in the presence of KOH in ethanol, but without NH_3_. In both cases, white precipitates formed within 5 min. The ESI mass spectra of the products were consistent with their being analogues of **6b**. Base peaks at *m*/*z* = 628.29 and 684.35 were assigned to [**6a** + H]^+^ and [**6c** + H]^+^, respectively (Figures S11 and S12). The ^1^H NMR spectra of **6a** and **6c** are shown in Figure 8a,c, and the similarity to that of **6b** (Figure 8b) is immediately apparent. The ^1^H and ^13^C NMR spectra of **6a** and **6c** (see Experimental Section) were assigned using routine 2D methods.

## 3. Review of Literature

### 3.1. Introduction

Although the Claisen-Schmidt condensation is presented as a simple reaction with a hydroxyketone intermediate and a single enone product in many chemistry text books, the reality is often far more complex. Although this paper is concerned with the use of acetylpyridines in the Claisen-Schmidt reaction, it is instructive to review the broader literature regarding the condensation of aromatic aldehydes with aromatic ketones.

**Figure 9:**
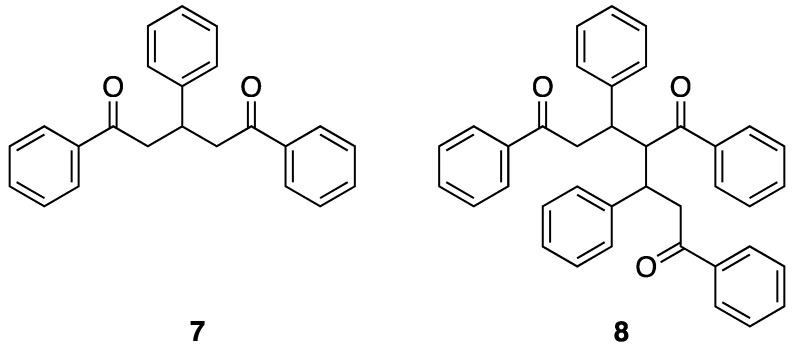
The structure of the 2:1 (ketone:aldehyde) pentane-1,5-dione that can arise from the reaction of acetophenone with benzaldehyde and the proposed structure of Kostanecki’s triketone the 3:2 adduct that formally arises from the nucleophilic addition of the enol of enolate of the pentane-1,5-dione on the 1:1 enone.

The enone products of the Claisen-Schmidt reaction are electrophilic and can react with nucleophilic enols or enolates at either the carbonyl carbon or by conjugate addition. The formation of the enone 1:1 products (1:1 ratio of ketone to aldehyde) involves the nucleophilic attack of an enol or enolate derived from the aromatic ketone upon the aromatic aldehyde. A number of publications have provided overviews of the reaction space and the types of products that are to be expected. If the rates of the subsequent reactions are significantly faster than the initial ones leading to the enone, domino [32,33,34] or tandem [35] reactions are expected, leading to the selective formation of one (or more) of a range of potential reaction products. In this section, we present a comprehensive overview of the products other than terpyridines that have been obtained from Kröhnke and related syntheses. We have already considered products of this type implicitly—the pentane-1,5-diones (**7**) arise from the addition of a second equivalent enol or enolate to the enone, giving products of 2:1 (ketone to aldehyde) stoichiometry (Figure 9).

### 3.2. The 3:2 Products

**Figure 10:**
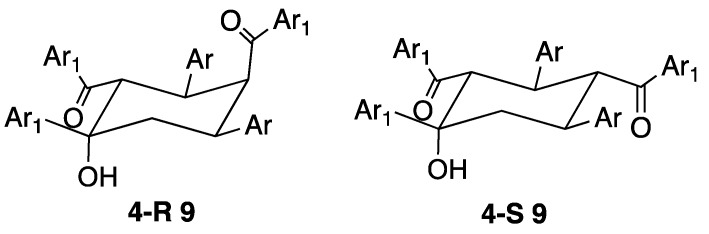
The structures of the 3:2 cyclohexanol derivatives arising from the cyclization of the triketones.

The most common of the products that have been isolated from attempted terpyridine syntheses have a reactant stoichiometry of 3:2 ketone-aldehyde. In one respect, this is an old story dating back to 1892 when the compound claimed to be Kostanecki’s triketone (**8**) was isolated as a product from the reaction of benzaldehyde and acetophenone [36]. Over the years, a number of materials purporting to be **8**, but possessing different physical properties have been reported [37,38]. A definitive report of the preparation and structural characterization of **8** describing the history of this compound together with an analysis of the various products isolated from the reaction of acetophenone with benzaldehyde appeared recently [39]. A large number of 3:2 condensation products have now been fully characterized by structural and spectroscopic means and shown to be cyclohexanols **9** arising from formally from the internal condensation of intermediate triketones. Naming the compounds as cyclohexanols for consistency (the IUPAC PIN is based upon 4-hydroxycyclohexane-1,3-diyl)bis(methanone)), the relative stereochemistry is 1R*, 2S*, 3R*, 5R*: the substituent in the 4-position may be either axial or equatorial giving both 4R* and 4S* (4-R* **9** or 4-S* **9,** Figure 10). The cyclic product is obtained in particularly high yield under phase transfer conditions from the reaction of benzaldehyde and acetophenone [40]. The reaction of enones with NaO*^t^*Bu and *N*-heterocyclic carbenes, yielded **9** and aldehyde [41]. A significant range of compounds with various Ar and Ar_1_ groups have been characterized, from one pot reactions of ArCHO and Ar_1_COCH_3_ or from sequential reactions of isolated enones. In addition to the parent compound (Ar = Ar_1_ = C_6_H_5_) [39,40,41,42,43,44,45,46], a large number of derivatives with carbocyclic Ar and Ar_1_ substituents have been obtained from reactions of substituted benzaldehydes and acetophenones [34,41,43,46,47,48,49,50,51,52,53]. Of most relevance to the chemistry reported in this manuscript, are compounds with heterocyclic substituents and, to date, examples with thiophen-2-yl [34,41,54,55,56], furan-2-yl [54,57], benzofuran-2-yl [33], benzofuran-3-yl [33], benzothiophen-2-yl [33], benzothiophen-3-yl [33], pyridin-2-yl [33,35,45,58,59,60,61,62,63,64,65,66] pyridin-3-yl [33,45,56] and pyridin-4-yl [16,20,33,45,56,67] groups have been reported. Cave and Raston have commented that reactions of 4-acetylpyridine with 4-alkoxybenzaldehydes in solution only yield the 3:2 products rather than the desired 4,2′:6′,4″-terpyridines [16,20]. We note, however, that in our hands and under the Hanan one-pot procedure [18], the 4′-(4-alkoxy)-4,2′:6′,4″-terpyridines shown in Figure 2 can be readily prepared [25].

To summarize, the formation of 3:2 condensation products is well-established with the stereochemistry at four of the five stereogenic centres defined. Examples of diastereoisomers with the 4-substituent in the axial or equatorial positions have been described and the less stable axial *R* diastereoisomer may be converted to the more stable equatorial *S* form with base [58].

### 3.3. The 3:1 Products

**Figure 11:**
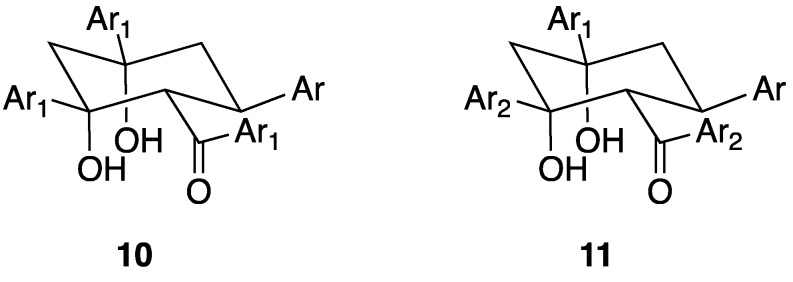
The structures of the 3:1 cyclohexane-1,3-diol derivatives arising from the condensation of the pentane-1,5-dione with Ar_1_COCH_3_.

A second type of cyclohexane derivative **10** (Figure 11) with a 3:1 (ketone-aldehyde) constitution is also known. We first described the formation of cyclohexane-1,3-diols from the condensation of 2-acetylpyridine with benzaldehyde derivatives in 1995 [58]. Although less commonly observed than the 3:2 condensation products, compounds of this type, arising from the aldol condensation of an intermediate pentane-1,5-dione with a third equivalent of ketone, have been isolated with a variety of substituents [32,60,68,69,70,71,72,73]. The relative stereochemistry in **10**, originally proposed on the basis of NMR studies [58], has been crystallographically confirmed [32,69,70,72]. Sequential reaction of the benzaldehyde derivative with two different ketones, Ar_1_COCH_3_ and Ar_2_COCH_3_ allows the synthesis of compounds **11** via the addition of Ar_2_COCH_3_ to the intermediate enone Ar_1_COCH=CHAr.

### 3.4. An Unexpected Terpyridine Isomer

**Scheme 3:**
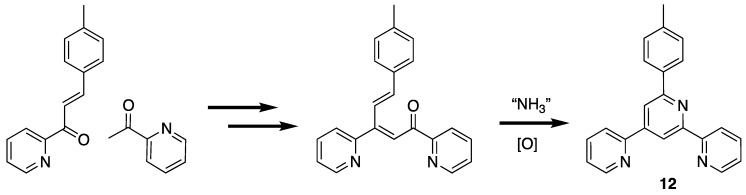
The origin of the isomeric 2,2′:4′,2″-terpyridine in 2,2′:6′,2″-terpyridine synthesis.

A third type of unexpected product obtained from attempted 4′-aryl-2,2′:6′,2″-terpyridine syntheses is the isomeric 6′-aryl-2,2′:4′,2″-terpyridine. This was first observed when 6′-(4-methylphenyl)-2,2′:4′,2″-terpyridine (**12**) was isolated as a side-product from the condensation of 2-acetylpyridine with 4-methylbenzaldehyde and ammonia [74,75,76,77,78,79,80]. This product presumably arises by the 1,2-attack of the enol or enolate of 2-acetylpyridine at the carbonyl (rather than the more usual 1,4-conjugate addition) of the intermediate enone, 3-(4-methylphenyl)-1-(pyridin-2-yl)prop-2-en-1-one (Scheme 3). Similarly, the preparation of 4′-phenyl-4,2′:6′,4″-terpyridine from 4-acetylpyridine and benzaldehyde gave appreciable amounts of 6′-(4-methylphenyl)-4,2′:4′,4″-terpyridine (**12**) [81].

## 4. Materials and Methods

^1^H and ^13^C and NMR spectra were recorded on a Bruker Avance iii-500 spectrometer (Bruker BioSpin AG, Fällanden, Switzerland) at 298 K. The ^1^H and ^13^C NMR chemical shifts were referenced with respect to residual solvent peaks (*δ* TMS = 0) and all quoted coupling constants *J* are *J*_HH_ between protons. A Shimadzu LCMS-2020 instrument (Shimadzu Schweiz GmbH, Roemerstr., Switzerland) was used to record electrospray ionization (ESI) mass spectra; samples were introduced as 200–800 μM solutions in MeCN with the addition of formic acid. PerkinElmer UATR Two (Perkin Elmer, Bahnstrasse 8, 8603 Schwerzenbach, Switzerland) and Cary-5000 (Agilent Technologies Inc., Santa Clara, CA, United States) spectrometers were used to record FT-infrared (IR) and absorption spectra, respectively. Melting points were measured using a Bibby Melting Point Apparatus SMP30.

3-Acetylpyridine was purchased from Acros Organics (Chemie Brunschwig AG, Basel, Switzerland), 4-ethoxybenzaldehyde and 4-butoxybenzaldehyde from Sigma Aldrich (Riedstr. 2, 89555 Steinheim, Germany), and 4-propoxybenzaldehyde from Fluorochem and were used as received.

### 4.1. Compound ***5a***

4-Ethoxybenzaldehyde (1.50 g, 1.39 mL, 10.0 mmol) was dissolved in EtOH (50 mL), then 3-acetylpyridine (2.42 g, 2.20 mL, 20.0 mmol) and crushed KOH (1.12 g, 20.0 mmol) were added to the solution. Aqueous NH_3_ (32%, 38.5 mL) was slowly added to the reaction mixture. This was stirred at room temperature overnight. The solid that formed was collected by filtration, washed with water (3 × 10 mL) followed by EtOH (3 × 10 mL), recrystallized from EtOH and dried *in vacuo*. Compound **5a** was isolated as a white powder (0.591 g, 1.67 mmol, 16.7%). M.p. = 120 °C. ^1^H NMR (500 MHz, CDCl_3_): *δ*/ppm = 9.37 (m, 2H, H^A2^), 8.70 (m, 2H, H^A6^), 8.51 (8.51 (dt, *J* = 8.0, 1.9 Hz, 2H, H^A4^), 7.91 (s, 2H, H^B3^), 7.70 (m, 2H, H^C2^), 7.47 (m, 2H, H^A5^), 7.05 (m, 2H, H^C3^), 4.12 (q, *J* = 7.0 Hz, 2H, H^a^), 1.47 (t, *J* = 7.0 Hz, 3H, H^b^). ^13^C{^1^H} NMR (500 MHz, CDCl_3_): *δ*/ppm = 160.1 (C^C4^), 155.0 (C^A3^), 150.3 (C^B4^), 149.7 (C^A6^), 148.0 (C^A2^), 134.8 (C^A4^), 134.6 (C^B2^), 130.0 (C^C1^), 128.2 (C^C2^), 123.6 (C^A5^), 117.1 (C^B3^), 115.1 (C^C3^), 63.6 (C^a^), 14.7 (C^b^). UV-VIS (CH_3_CN, 3.3 × 10^–5^ mol dm^–3^) λ/nm 229 (ε/dm^3^ mol^–1^ cm^–1^ 29,700), 272 (33,800). ESI-MS *m*/*z* 354.08 [M + H]^+^ (calc. 354.16). Found C 77.38, H 5.30, N 11.91; required for C_23_H_19_N_3_O: C 78.16, H 5.42, N 11.89.

### 4.2. Compound ***5c***

4-Butoxybenzaldehyde (1.78 g, 1.73 mL, 10.0 mmol) was dissolved in EtOH (50 mL), then 3-acetylpyridine (2.42 g, 2.20 mL, 20.0 mmol) and crushed KOH (1.12 g, 20.0 mmol) were added to the solution. Aqueous NH_3_ (32%, 38.5 mL) was slowly added to the reaction mixture. This was stirred at room temperature overnight. The solid that formed was collected by filtration, washed with water (3 × 10 mL) and EtOH (3 × 10 mL), then recrystallized from EtOH and dried *in vacuo*. Compound **5c** was isolated as a white powder (1.19 g, 3.11 mmol, 31.1%). M.p. = 113 °C. ^1^H NMR (500 MHz, CDCl_3_): *δ*/ppm = 9.39 ((d, *J* = 2.2 Hz, 2H, H^A2^), 8.72 ((dd, *J* = 4.9, 1.6 Hz, 2H, H^A6^), 8.59 (dt, *J* = 8.1, 2.0 Hz, 2H, H^A4^), 7.95 (s, 2H, H^B3^), 7.71 (m, 2H, H^C2^), 7.53 (m, 2H, H^A5^), 7.07 (m, 2H, H^C3^), 4.06 (t, *J* = 6.5 Hz, 2H, H^a^), 1.83 (m, 2H, H^b^), 1.54 (m, 2H, H^c^), 1.01 (t, *J* = 7.4 Hz, 3H, H^d^). ^13^C{^1^H} NMR (500 MHz, CDCl_3_): *δ*/ppm = 160.8 (C^C4^), 154.9 (C^A3^), 150.9 (C^B4^), 149.0 (C^A6^), 147.4 (C^A2^), 135.8 (C^A4^), 135.4 (C^B2^), 129.9 (C^C1^), 128.5 (C^C2^), 124.2 (C^A5^), 117.6 (C^B3^), 115.5 (C^C3^), 68.1 (C^a^), 31.4 (C^b^), 19.4 (C^c^), 14.0 (C^d^). UV-VIS (CH_3_CN, 3.3 × 10^–5^ mol dm^–3^) λ/nm 228 (ε/dm^3^ mol^–1^ cm^–1^ 30,100), 274 (33,700). ESI-MS *m*/*z* 382.16 [M + H]^+^ (calc. 382.19). Found C 78.37, H 5.93, N 11.05; required for C_25_H_23_N_3_O: C 78.71, H 6.08, N 11.02.

### 4.3. Compound ***6b***: Method 1

4-Propoxybenzaldehyde (1.64 g, 1.58 mL, 10.0 mmol) was dissolved in EtOH (50 mL), then 3-acetylpyridine (2.42 g, 2.20 mL, 20.0 mmol) and crushed KOH (1.12 g, 20.0 mmol) were added to the solution. At this stage, a white precipitate began to form. Aqueous NH_3_ (32%, 38.5 mL) was slowly added to the reaction mixture which was then stirred at room temperature overnight. The solid that formed was collected by filtration, washed with water (3 × 10 mL) then with EtOH (3 × 10 mL), and was recrystallized from EtOH and dried *in vacuo*. Compound **6b** was isolated as a white powder (0.495 g, 0.755 mmol, 15.1%). M.p. = 208 °C. ^1^H NMR (500 MHz, CDCl_3_): *δ/*ppm = 9.04 (d, *J* = 1.9 Hz, 1H, H^C2^), 8.75 (d, *J* = 1.8 Hz, 1H, H^B2^), 8.52 (dd, *J* = 4.8, 1.6 Hz, 1H, H^B6^), 8.42 (dd, *J* = 4.8, 1.6 Hz, 1H, H^A6^), 8.38 (dd, *J* = 4.8, 1.5 Hz, 1H, H^C6^), 8.34 (d, *J* = 1.7 Hz, 1H, H^A2^), 8.11 (dt, *J* = 8.1, 1.8 Hz, 1H, H^C4^), 7.71 (dt, *J* = 8.1, 2.0 Hz, 1H, H^B4^), 7.42 (dt, *J* = 8.1, 2.0 Hz, 1H, H^A4^), 7.25 (m, 1H, H^C5^), 7.11 (m, 1H, H^B5^), 7.06 (m, 2H, H^D2^), 7.01 (m, 1H, H^A5^), 6.95 (m, 2H, H^E2^), 6.61 (m, 2H, H^D3^), 6.39 (m, 2H, H^E3^), 5.62 (d, *J* = 11.9 Hz, 1H, H^2^), 5.08 (d, *J* = 2.1 Hz, 1H, H^OH^), 4.29 (dd, *J* = 4.7, 4.7 Hz, 1H, H^4^), 4.08 (m, 2H, H^3+5^), 3.73 (m, 2H, H^a′^), 3.60 (m, 2H, H^a^), 3.20 (ddd, *J* = 13.0, 13.0, 2.5 Hz, 1H, H^6ax^), 2.03 (dd, *J* = 13.7, 3.4 Hz, 1H, H^6eq^), 1.67 (m, 2H, H^b′^), 1.59 (m, 2H, H^b^), 0.94 (t, *J* = 7.4 Hz, 3H, H^c′^), 0.87 (t, *J* = 7.4 Hz, 3H, H^c^). ^13^C{^1^H} NMR (500 MHz, CDCl_3_): *δ/*ppm = 206.8 (C^CO2^), 206.1 (C^CO1^), 158.3 (C^E4^), 158.1 (C^D4^), 153.7 (C^B6^), 152.5 (C^A6^), 149.6 (C^B2^), 149.1 (C^A2^), 147.9 (C^C6^), 146.4 (C^C2^), 142.6 (C^C3^), 135.2 (C^B4^), 135.0 (C^A3^), 134.8 (C^A4^), 133.8 (C^C4^), 133.0 (C^B3^), 132.7 (C^D1^), 130.2 (C^E1^), 129.7 (C^E2^), 128.6 (C^D2^), 123.5 (C^C5^), 123.2 (C^B5^), 122.9 (C^A5^), 114.7 (C^D3^), 114.6 (C^E3^), 75.0 (C^1^), 69.6 (C^a′^), 69.4 (C^a^), 53.5 (C^4^), 51.0 (C^2^), 46.8 (C^3^), 41.2 (C^5^), 38.8 (C^6^), 22.6 (C^b′^), 22.5 (C^b^), 10.6 (C^c′^), 10.5 (C^c^). UV-VIS (CH_3_CN, 3.3 × 10^–5^ mol dm^–3^) λ/nm 228 (ε/dm^3^ mol^–1^ cm^–1^ 38,100), 264 (10,400). ESI-MS *m/z* 656.31 [M + H]^+^ (calc. 656.31). IR spectrum: see Figure S6. Found C 74.67, H 6.77, N 6.73; required for C_41_H_41_N_3_O_5_ C 75.09, H 6.30, N 6.41.

### 4.4. Compound ***6b***: Method 2

The reaction was carried out as in method 1 without the addition of NH_3_. Reagents and solvent: 4-propoxybenzaldehyde (0.82 g, 0.79 mL, 5.0 mmol), EtOH (25 mL), 3-acetylpyridine (1.21 g, 1.10 mL, 10.0 mmol) and crushed KOH (0.56 g, 10.0 mmol). **6b** was isolated as a white powder (0.690 g, 1.05 mmol, 42.0%). Characterization data matched those for the product reported in Section 4.3.

### 4.5. Compound ***6a***

4-Ethoxybenzaldehyde (0.75 g, 0.70 mL, 5.0 mmol) was dissolved in EtOH (25 mL), then 3-acetylpyridine (1.21 g, 1.10 mL, 10.0 mmol) and crushed KOH (0.56 g, 10.0 mmol) were added to the solution. After five minutes a white precipitate began to form. The reaction mixture was stirred at room temperature overnight. The solid that formed was collected by filtration, washed with water (3 × 10 mL) then EtOH (3 × 10 mL), recrystallized from EtOH and dried in vacuo. **6a** was isolated as a white powder (0.268 g, 0.427 mmol, 17.1%). M.p. = 210 °C. ^1^H NMR (500 MHz, CDCl_3_): *δ/*ppm = 9.06 (d, *J* = 1.9 Hz, 1H, H^C2^), 8.76 (d, *J* = 1.8 Hz, 1H, H^B2^), 8.54 (dd, *J* = 4.8, 1.6 Hz, 1H, H^B6^), 8.44 (dd, *J* = 4.8, 1.6 Hz, 1H, H^A6^), 8.41 (dd, *J* = 4.9, 1.3 Hz, 1H, H^C6^), 8.34 (d, *J* = 1.8 Hz, 1H, H^A2^), 8.25 (m, 1H, H^C4^), 7.72 (dt, *J* = 8.0, 1.9 Hz, 1H, H^B4^), 7.43 (dt, *J* = 8.0, 1.9 Hz, 1H, H^A4^), 7.37 (m, 1H, H^C5^), 7.13 (m, 1H, H^B5^), 7.05 (m, 2H, H^D2^), 7.02 (m, 1H, H^A5^), 6.95 (m, 2H, H^E2^), 6.61 (m, 2H, H^D3^), 6.39 (m, 2H, H^E3^), 5.62 (d, *J* = 11.9 Hz, 1H, H^2^), 5.18 (d, *J* = 2.1 Hz, 1H, H^OH^), 4.29 (dd, *J* = 4.6, 4.6 Hz, 1H, H^4^), 4.08 (m, 2H, H^3+5^), 3.85 (m, 2H, H^a′^), 3.71 (m, 2H, H^a^), 3.19 (ddd, *J* = 13.6, 13.6, 2.1 Hz, 1H, H^6ax^), 2.04 (dd, *J* = 13.7, 3.4 Hz, 1H, H^6eq^), 1.29 (t, *J* = 7.0 Hz, 3H, H^b′^), 1.20 (t, *J* = 7.0 Hz, 3H, H^b^). ^13^C{^1^H} NMR (500 MHz, CDCl_3_): *δ/*ppm = 206.6 (C^CO2^), 206.1 (C^CO1^), 158.2 (C^E4^), 158.0 (C^D4^), 153.9 (C^B6^), 152.6 (C^A6^), 149.6 (C^B2^), 149.1 (C^A2^), 146.2 (C^C6^), 144.8 (C^C2^), 143.2 (C^C3^), 135.3 (C^C4^), 134.8 (C^A3^), 134.9 (C^B4^), 134.8 (C^A4^), 132.8 (C^B3^), 132.6 (C^D1^), 130.1 (C^E1^), 129.7 (C^E2^), 128.6 (C^D2^), 123.5 (C^C5^), 123.4 (C^B5^), 123.0 (C^A5^), 114.7 (C^D3^), 114.6 (C^E3^), 75.1 (C^1^), 63.5 (C^a′^), 63.2 (C^a^), 53.4 (C^4^), 50.8 (C^2^), 46.9 (C^3^), 41.2 (C^5^), 38.9 (C^6^), 14.8 (C^b′^), 14.7 (C^b^). ESI-MS m/z 628.29 [M + H]^+^ (calc. 628.28). IR spectrum: see Figure S13. Found C 74.26, H 5.88, N 6.53; required for C_39_H_37_N_3_O_5_ C 74.62, H 5.94, N 6.69.

### 4.6. Compound ***6c***

4-Butoxybenzaldehyde (0.89 g, 0.87 mL, 5.0 mmol) was dissolved in EtOH (25 mL), then 3-acetylpyridine (1.21 g, 1.10 mL, 10.0 mmol) and crushed KOH (0.56 g, 10.0 mmol) were added to the solution. After five minutes a white precipitate began to form. The reaction mixture was stirred at room temperature overnight. The precipitate was collected by filtration, washed with water (3 × 10 mL) and EtOH (3 × 10 mL), then recrystallized from EtOH and dried in vacuo. **6c** was isolated as a white powder (0.399 g, 0.585 mmol, 11.7%). M.p. = 185 °C. ^1^H NMR (500 MHz, CDCl_3_): *δ/*ppm = 9.06 (d, *J* = 1.8 Hz, 1H, H^C2^), 8.77 (d, *J* = 1.8 Hz, 1H, H^B2^), 8.54 (dd, *J* = 4.8, 1.6 Hz, 1H, H^B6^), 8.43 (dd, *J* = 4.8, 1.7 Hz, 1H, H^A6^), 8.42 (dd, *J* = 5.0, 1.4 Hz, 1H, H^C6^), 8.35 (d, *J* = 1.7 Hz, 1H, H^A2^), 8.27 (m, 1H, H^C4^), 7.72 (dt, *J* = 8.0, 1.9 Hz, 1H, H^B4^), 7.44 (dt, *J* = 8.0, 2.0 Hz, 1H, H^A4^), 7.39 (m, 1H, H^C5^), 7.13 (m, 1H, H^B5^), 7.05 (m, 2H, H^D2^), 7.02 (m, 1H, H^A5^), 6.95 (m, 2H, H^E2^), 6.61 (m, 2H, H^D3^), 6.39 (m, 2H, H^E3^), 5.63 (d, *J* = 11.9 Hz, 1H, H^2^), 5.18 (d, *J* = 2.1 Hz, 1H, H^OH^), 4.29 (dd, *J* = 4.6, 4.6 Hz, 1H, H^4^), 4.08 (m, 2H, H^3+5^), 3.77 (m, 2H, H^a′^), 3.64 (m, 2H, H^a^), 3.18 (ddd, *J* = 13.7, 13.7, 2.0 Hz, 1H, H^6ax^), 2.04 (dd, *J* = 13.7, 3.4 Hz, 1H, H^6eq^), 1.63 (m, 2H, H^b′^), 1.55 (m, 2H, H^b^), 1.39 (m, 2H, H^c′^), 1.32 (m, 2H, H^c^), 0.91 (t, *J* = 7.4 Hz, 3H, H^d′^), 0.86 (t, *J* = 7.4 Hz, 3H, H^d^). ^13^C{^1^H} NMR (500 MHz, CDCl_3_): *δ/*ppm = 207.4 (C^CO2^), 206.8 (C^CO1^), 158.3 (C^D4^), 158.1 (C^E4^), 153.9 (C^B6^), 152.4 (C^A6^), 149.4 (C^B2^), 149.0 (C^A2^), 145.9 (C^C6^), 144.5 (C^C2^), 143.8 (C^C3^), 135.6 (C^C4^), 134.9 (C^A3^), 135.2 (C^B4^), 134.8 (C^A4^), 132.9 (C^B3^), 132.3 (C^D1^), 130.0 (C^E1^), 129.7 (C^E2^), 128.5 (C^D2^), 124.1 (C^C5^), 123.3 (C^B5^), 122.7 (C^A5^), 114.8 (C^D3^), 114.6 (C^E3^), 75.4 (C^1^), 67.7 (C^a′^), 67.5 (C^a^), 53.3 (C^4^), 50.6 (C^2^), 46.7 (C^3^), 41.2 (C^5^), 38.8 (C^6^), 31.3 (C^b′^), 31.2 (C^b^), 19.3 (C^c′^), 19.2 (C^c^), 13.8 (C^d′^), 13.7 (C^d^). ESI-MS *m*/*z* 684.35 [M + H]^+^ (calc. 684.34). IR spectrum: see Figure S14. Found C 75.31, H 6.67, N 6.11; required for C_43_H_45_N_3_O_5_ C 75.52, H 6.63, N 6.14.

## 5. Conclusions

The preparation of terpyridines from the reactions of acetylpyridines with aromatic aldehydes and ammonia is an established synthetic method. Nevertheless, the reaction can give a variety of products. This paper provides another example of an ″unexpected″ product and a systematic survey of the products of such reactions. Although the one-pot synthesis of terpyridines is presented in the literature as an infallible synthetic method, there is ample precedent for the formation of a variety of alternative products. In particular, the assumption that the material precipitating from the reaction mixture is the desired terpyridine is not always correct. As these reactions are commonly used in the coordination chemistry community, this paper serves as a useful caveat. We are currently further investigating these reactions with varying length alkyloxy chains and will report on the constitution of the full reaction space in the future.

## Supplementary Materials

The following are available online. Figures S1 and S2: Mass spectra of **5a** and **5c**; **Figure S3**: ^13^C{^1^H} NMR spectra of **5a** and **5c**; Figures S4–S6: IR spectra of **5a**, **5c** and **6b**; Figure S7: Mass spectrum of **6b**; Figures S8–S10: NOESY, HMQC and HMBC spectra of **6b**; Figures S11 and S12: Mass spectra of **6a** and **6c**; Figures S3 and S4: IR spectra of **6a** and **6c**.

## References

[1] Favre H.A., Powell W.H. (2013). Nomenclature of Organic Chemistry. IUPAC Recommendations and Preferred Names 2013.

[2] Schubert U.S., Hofmeier H., Newkome G.R. (2006). Modern Terpyridine Chemistry.

[3] Schubert U.S., Winter A., Newkome G.R. (2011). Terpyridine-Based Materials: For Catalytic, Optoelectronic and Life Science Applications.

[4] Constable E.C. (2007). 2,2′:6′,2″-Terpyridines: From chemical obscurity to common supramolecular motifs. Chem. Soc. Rev..

[5] Wei C., He Y., Shi X., Song Z. (2019). Terpyridine-metal complexes: Applications in catalysis and supramolecular chemistry. Coord. Chem. Rev..

[6] Constable E.C. (1994). Higher Oligopyridines as a Structural Motif in Metallosupramolecular Chemistry. Progr. Inorg. Chem..

[7] Constable E.C. (1986). The Coordination Chemistry of 2,2′:6′,2″-Terpyridine and Higher Oligopyridines. Adv. Inorg. Chem..

[8] Favre H.A., Powell W.H. (2013). Nomenclature of Organic Chemistry. IUPAC Recommendations and Preferred Names 2013.

[9] Kröhnke F. (1976). The Specific Synthesis of Pyridines and Oligopyridines. Synthesis.

[10] Kostanecki St. von, Tambor J. (1899). Über die sechs isomeren Monooxybenzalacetophenone (Monooxychalkone). Chem. Ber..

[11] Wang Z. (2010). Comprehensive Organic Name Reactions and Reagents.

[12] Heathcock C.H., Knochel P., Molander G.A. (2014). The Aldol Reaction: Group I and Group II Enolates in Comprehensive Organic Synthesis II.

[13] Ortoleva G. (1900). Azione del jodio sull’acido malonico in soluzione piridica. Gazz. Chim. Ital..

[14] King L.C. (1944). The reaction of iodine with some ketones in the presence of pyridine. J. Am. Chem. Soc..

[15] Kröhnke F. (1963). Syntheses Using Pyridinium Salts. Angew. Chem. Int. Ed..

[16] Cave G.W.V., Raston C.L. (2000). Toward benign syntheses of pyridines involving sequential solvent free aldol and Michael addition reactions. Chem. Commun..

[17] Smith N.M., Raston C.L., Smith C.B., Sobolev A.N. (2007). PEG mediated synthesis of amino-functionalised 2,4,6-triarylpyridines. Green Chem..

[18] Wang J., Hanan G.S. (2005). A facile route to sterically hindered and non-hindered 4′-aryl-2,2′:6′,2″- terpyridines. Synlett.

[19] Cooke M.W., Wang J., Theobald I., Hanan G.S. (2006). Convenient One-Pot Procedures for the Synthesis of 2,2′:6′,2″-Terpyridine. Synth. Commun..

[20] Cave G.W.V., Raston C.L. (2001). Efficient synthesis of pyridines via a sequential solventless aldol condensation and Michael addition. J. Chem. Soc. Perkin Trans. 1.

[21] Housecroft C.E. (2014). 4,2′:6′,4″-Terpyridines: Diverging and diverse building blocks in coordination polymers and metallomacrocycles. Dalton Trans..

[22] Housecroft C.E. (2015). Divergent 4,2′:6′,4″- and 3,2′:6′,3″-terpyridines as linkers in 2- and 3-dimensional architectures. CrystEngComm.

[23] Constable E.C., Housecroft C.E. (2018). Tetratopic bis(4,2′:6′,4″-terpyridine) and bis(3,2′:6′,3″-terpyridine) ligands as 4-connecting nodes in 2D-coordination networks and 3D-frameworks. J. Inorg. Organomet. Polym. Mater..

[24] Housecroft C.E., Constable E.C. (2019). Ditopic and tetratopic 4,2′:6′,4″-Terpyridines as Structural Motifs in 2D- and 3D-Coordination Assemblies. Chimia.

[25] Klein Y.M., Constable E.C., Housecroft C.E., Zampese J.A., Crochet A. (2014). Greasy tails switch 1D-coordination [{Zn_2_(OAc)(4′-(4-ROC_6_H_4_)-4,2′:6′,4″-tpy)}*_n_*] polymers to discrete[Zn_2_(OAc)(4′-(4-ROC_6_H_4_)-4,2′:6′,4″-tpy)_2_] complexes. CrystEngComm.

[26] Klein Y.M., Constable E.C., Housecroft C.E., Prescimone A. (2014). Assembling coordination ladders with 4′-(4-methoxyphenyl)- 4,2′:6′,4″-terpyridine as rails and rungs. Inorg. Chem. Commun..

[27] Constable E.C., Zhang G., Housecroft C.E., Zampese J.A. (2012). A matter of greasy tails: Interdigitation of alkyl chains in free and coordinated 4′-(4-dodecyloxyphenyl)-4,2′:6′,4″-terpyridines. Inorg. Chem. Commun..

[28] Klein Y.M., Prescimone A., Constable E.C., Housecroft C.E. (2015). Manipulating connecting nodes through remote alkoxy chain variation in coordination networks with 4′-alkoxy-4,2′:6′,4″-terpyridine linkers. CrystEngComm.

[29] Klein Y.M., Prescimone A., Constable E.C., Housecroft C.E. (2016). 2-Dimensional networks assembled using 4′-functionalized 4,2′:6′,4″-terpyridines and Co(NCS)_2_. Polyhedron.

[30] Klein Y.M., Prescimone A., Pitak M.B., Coles S.J., Constable E.C., Housecroft C.E. (2016). Constructing chiral MOFs by functionalizing 4,2′:6′,4″-terpyridine with long-chain alkoxy domains: Rare examples of *neb* nets. CrystEngComm.

[31] Li L., Zhang Y.Z., Yang C., Liu E., Golen J.A., Zhang G. (2016). One-dimensional copper(II) coordination polymers built on 4′-substituted 4,2′:6′,4″ and 3,2′:6′,3″-terpyridines: Syntheses, structures and catalytic properties. Polyhedron.

[32] Chang M.-Y., Wu M.-H. (2012). Domino cyclocondensation of arylaldehydes with 2-acetylpyridine. Tetrahedron.

[33] Patel P.N., Chadha A. (2018). A simple metal free highly diastereoselective synthesis of heteroaryl substituted (±) cyclohexanols by a branched domino reaction. Tetrahedron.

[34] Gezegen H., Ceylan M. (2015). Alternate Method for the Synthesis of Six-Membered Carbocycles with Five Stereocenters:1,2,3,4,6-Pentasubstituted-4-hydroxy-cyclohexanes. Synth. Commun..

[35] Li C.-W., Shen T.-H., Shih T.L. (2017). Reinvestigation of synthesis of halo-substituted 3-phenyl-1-(2-pyridyl)-2-propen-1-ones (azachalcones). A tandem reaction for formation of penta-substituted cyclohexanols. Tetrahedron.

[36] Kostanecki S.V., Rossbach G. (1896). Ueber die Einwirkung von Benzaldehyd auf Acetophenon. Ber. Dtsch. Chem. Ges..

[37] Hodnett E.M., Ross W.W. (1951). Condensations of Aldehydes and Ketones Catalyzed by Potassium Cyanide. Proc. Oklahoma Acad. Sci..

[38] Georgi R., Schwyzer A. (1913). Versuche, d-Fenchon oder Campher an Benzalacetophenon oder an andere α,β-ungesättigte Ketone zu addieren. J. Prakt. Chem..

[39] Shan Z., Hu X., Hu L., Peng X. (2009). First Authentication of Kostanecki′s Triketone and Multimolecular Reaction of Aromatic Aldehydes with Acetophenone. Helv. Chim. Acta.

[40] Inoue K., Noguchi H., Hidai M., Uchida Y. (1983). Synthesis of a novel carbon ring compound, 2,4-dibenzoyl-1,3,5-triphenylcyclohexanol, from acetophenone and benzaldehyde under phase transfer conditions. Yukagaku (J. Japan Oil Chem. Soc.).

[41] Zhang Y., Wu X., Hao L., Wong Z.R., Lauw S.J.L., Yang S., Richard D., Webster R.D., Chi Y.R. (2017). Trimerization of enones under air enabled by NHC/NaOtBu via a SET radical pathway. Org. Chem. Front..

[42] Vasileyev B.K., Bagrina N.P., Vysotskii V.I., Lindeman S.V., Struchkov Y.T. (1990). Structure of Kostanecki’s Triketone. Acta Crystallogr. Sect. C Cryst. Struct. Commun..

[43] Minyaev M.E., Roitershtein D.M., Nifant’ev I.E., Ananyev I.V., Minyaeva T.V., Mikhaylyev T.A. (2015). A structural study of (1RS,2SR,3RS,4SR,5RS)-2,4-dibenzoyl-1,3,5-tri phenyl cyclo hexan-1-ol chloro form hemisolvate and (1RS,2SR,3RS,4SR,5RS)-2,4-dibenzoyl-1-phenyl-3,5-bis(2-methoxyphenyl)cyclohexan-1-ol. Acta Crystallogr. Sect. C: Struct. Chem..

[44] Zhang J.-H., He Q.-P., Wang Y., Wang D.-Q. (2007). 2,4-Di benzoyl-1,3,5-tri phenyl cyclo hexan-1-ol di chloromethane hemisolvate. Acta Crystallogr. Sect. E Struct. Rep. Online.

[45] Yin Y.G., Cheung K.K., Wong W.T. (1998). One-pot Synthesis of Substituted Cyclohexanes from Condensation of Aldehyde and Methylketone. Chin. Chem. Lett..

[46] Rong L., Wei X., Lu Y., Zong Z. (2012). A Facile and Efficient Synthesis of Polysubstituted Cyclohexanol Derivatives under Solvent-Free Conditions. Chin. J. Org. Chem..

[47] Mamedov I., Abbasoglu R., Bayramov M., Maharramov A. (2016). Synthesis of a new 1,2,3,4,5-pentasubstituted cyclohexanol and determining its stereochemistry by NMR spectroscopy and quantum-chemical calculations. Magn. Reson. Chem..

[48] Luo X., Shan Y. (2006). 2,4-Dibenzoyl-3,5-bis (4-methoxylphenyl)-1-phenyl cyclohexanol. Acta Crystallogr. Sect. E Struct. Rep. Online.

[49] Mukhtar S., Alsharif M.A., Alahmdi M.I., Parveen H. (2018). Synthesis, Characterization, Stereochemistry and Biological Evaluation of Novel Cyclohexanol Derivatives. Asian J. Chem..

[50] Chen W.-Y., Peng Y.-K. (2000). Microwave irradiated synthesis of substituted cyclohexanes. Hecheng Huaxue.

[51] Hussain H.T., Osama M., Hussain W. (2014). Stereostructure, Antimicrobial and Cytotoxic Activity of Cyclohexene, Cyclohexanol and Pyridine Derivatives Synthesized from Chalcones. Int. J. Pharm. Sci. Res..

[52] Shan Z., Luo X., Hu L., Hu X.-Y. (2010). New observation on a class of old reactions: Chemoselectivity for the solvent-free reaction of aromatic aldehydes with alkylketones catalyzed by a double-component inorganic base system. Sci. China Chem..

[53] Luo X., Shan Z. (2006). Highly chemoselective synthesis of 1,2,3,4,5-pentasubstituted cyclohexanols under solvent-free condition. Tetrahedron Lett..

[54] Çelik I., Ersanl C.C., Akkurt M., Gezegen H., Köseoğlu R. (2016). Crystal structure of racemic [(1R,2S,3R,4S,6S)-2,6-bis-(furan-2-yl)-4-hydroxy-4-(thiophen-2-yl)cyclohexane-1,3-diyl]bis(thiophen-2-yl methanone). Acta Crystallogr. Sect. C Struct. Chem..

[55] Wang X.-F., Huang X.-Q. (2008). 2,4-Bis(4-chlorobenzoyl)-1-(4-chlorophenyl)-3,5-di-2-thienylcyclohexanol methanol hemisolvate. Acta Crystallogr. Sect. E Struct. Rep. Online..

[56] Vatsadze S.Z., Nuriev V.N., Leshcheva I.F., Zyk N.V. (2004). New aspects of the aldol condensation of acetylpyridines with aromatic aldehydes. Russ. Chem. Bull. Int. Ed..

[57] Chen X.-M., Yin Y.-G., Chen H.-R., Ding J. (2006). One-pot Synthesis and Crystal Structure of (1S,2R,3S,4R,5S)-1-Phenyl-2,4-dibenzoyl-3,5-difurylhexanol. Chin. J. Struct. Chem..

[58] Thompson A.M.W.C., Constable E.C., Harverson P., Phillips D., Raithby P.R., Powell H.R., Ward M.D. (1995). Condensation Reactions of 2-Acetylpyridine with Benzaldehydes—The Synthesis and Characterization of Some Cyclohexanols and Cyclohexanediols. J. Chem. Res. (S).

[59] Constable E.C., Zhang G., Housecroft C.E., Neuburger M., Schaffner S. (2009). Phase-separated hydrogen-bonded chloride ion–water–oxonium ion sheets and protonated 4′-(4-bromophenyl)-2,2:6′,2″-terpyridine stacks, and condensation products of 2-acetylpyridine and benzaldehydes revisited. CrystEngComm.

[60] Korall P., Börje A., Norrby P.-O., Åkermark B. (1997). High Yield Preparation of 4′-(4-Bromophenyl)-2,2′: 6′,2″-terpyridine by a Condensation Reaction. Determination of the Stereochemistry of Two Complex By-products by a Combination of Molecular Mechanics and NMR Spectroscopy. Acta Chem. Scand..

[61] Samshuddin S., Jasinski J.P., Butcher R.J., Neuhardt E.A., Narayana B., Yathirajan H.S., Glidewell C. (2014). Three closely-related cyclo hexanols (C_35_H_27_X_2_N_3_O_3_; X = F, Cl or Br): Similar molecular structures but different crystal structures. Acta Crystallogr. Sect. C Struct. Chem..

[62] Krishnapriya K.R., Sampath N., Aravindhan S., Ponnuswamy M.N., Kandaswamy M. (2004). 2,4-Bis (pyridine-2-carbonyl)-1-(2-pyridyl)-3,5-di-ptolylcyclohex-1-ol. Acta Crystallogr. Sect. E Struct. Rep. Online..

[63] Fun H.-K., Ooi C.W., Samshuddin S., Narayanan B., Sarojini B.K. (2012). [2,6-Bis(biphenyl-4-yl)-4-hydroxy-4-(pyridin-2-yl)cyclohexane-1,3-diyl]bis[(pyridin-2-yl)methanone]–butan-2-one (1/1). Acta Crystallogr. Sect. E Struct. Rep. Online.

[64] Downs L.E., Wolfe D.M., Schreiner P.R. (2005). Organic Base-Mediated Condensation of Pyridinecarboxaldehydes to Azachalcones. Adv. Synth. Catal..

[65] Fry D., Huang K.S., Di Lello P., Mohr P., Müller K., So S.-S., Harada T., Stahl M., Vu B., Mauser H. (2013). Design of Libraries Targeting Protein–Protein Interfaces. ChemMedChem.

[66] Chamchoumis C., Potvin P. (1998). Condensation Reactions of 2-Acetylpyridine and Benzaldehydes: New Cyclohexanol Products and an Improved Synthesis of 4′-p-Tolyl-2,2′ :6′,2″-terpyridine. J. Chem. Res. (S).

[67] Kessler H., Mronga S., Kutscher B., Miiller A., Sheldrick W.S. (1991). Synthesis of the 4-Pyridine Analog of Kostanecki’s Triketone. Determination of Constitution and Stereochemistry by 2 D-NMR Spectroscopy and X-ray Structural Analysis. Liebigs Ann. Chem..

[68] Wang H., Chen Y., Ye W., Xu J., Liu D., Yang J., Kong L., Zhou H., Tian Y., Tao Y. (2013). A facile and highly efficient green synthesis of carbazole derivatives containing a six-membered ring. Dyes Pigm..

[69] Ge X., Hao F., Li S., Jin F., Zhou H. (2014). Synthesis and Crystal Structure of New Pyridyl Derivative. Asian J. Chem..

[70] Zhang Y., Pan J., Zhang C., Wang H., Zhang G., Kong L., Tian Y., Yang J. (2015). High quantum yield both in solution and solid state based on cyclohexyl modified triphenylamine derivatives for picric acid detection. Dyes Pigm..

[71] Constable E.C., Harverson P., Smith D.R., Whall L. (1997). The coordination chemistry of 4′-(4′-tertbutylphenyl)-2,2′:6′,2″-terpyridine-a solubilising oligopyridine. Polyhedron.

[72] Constable E.C., Handel R., Housecroft C.E., Neuburger M., Schofield E.R., Zehnder M. (2004). Efficient syntheses of 4′-(2-thienyl)- and 4′-(3-thienyl)-2,2′:6′,2″-terpyridine: Preparation and characterization of Fe(II), Ru(II), Os(II) and Co(II) complexes. Polyhedron.

[73] Li L., Yang J.-X., Tian Y.-P., Liu P., Jin B.-K., Tao X.-T., Min-Hua Jiang M.-H. (2008). Synthesis, characterization and crystal structure of 6-ferrocenyl-2,4-dihydroxy-2,4-di(pyridine-2-yl)cyclohexanecarbonyl ferrocene. Transit. Met. Chem..

[74] Bray D.J., Clegg J.K., Jolliffe K.A., Lindoy L.F., Wei G. (2008). Synthesis and co-crystallisation behaviour of copper(II) complexes of two isomeric p-tolyl-terpyridines. J. Coord. Chem..

[75] Collin J.-P., Guillerez S., Sauvage J.-P., Barigelletti F., De Cola L., Flamigni L., Balzani V. (1991). Photoinduced processes in dyads and triads containing a ruthenium (II)-bis (terpyridine) photosensitizer covalently linked to electron donor and acceptor groups. Inorg. Chem..

[76] Turonek M.L., Moore P., Errington W. (2000). Synthesis of the Terpyridyl Pendant-arm Azamacrocycle 4′-(p-1,4,7- triazacyclonon-1-ylmethylphenyl)-2,2′:6′,2″-terpyridine (L) and Complexes of L with Copper(II) and Nickel(II). Crystal Structure of [Cu(HL)(H_2_O)_2_][PF_6_]_3_. J. Chem. Soc. Dalton Trans..

[77] Jing B.-W., Wu T., Zhang M.-H., Shen T. (2000). Synthesis of the Polypyridine Ligands with Functional Groups. Gaodeng Xuexiao Huaxue Xuebao (Chem. J. High. Educ. Inst.).

[78] Shirai H., Hanabusa K., Takahashi M.F., Hanada K. (1996). Neurodegenerative disease therapeutics containing dipyridyltolylpyridine. Jpn. Kokai Tokkyo Koho.

[79] Rajalakshmanan E., Alexander V. (2007). Synthesis, Luminescence, and Electrochemical Studies of Tris(homoleptic) Ruthenium(II) and Osmium(II) Complexes of 6′-Tolyl-2,2′:4′,2″-terpyridine. Inorg. Chem..

[80] Zhang D.-Y., Nie Y., Sang H., Suo J.-J., Li Z.-J., Gu W., Tian J.-L., Liu X., Yan S.-P. (2017). Three structurally related Copper complexes with two isomers: DNA/BSA binding ability, DNA cleavage activity and excellent cytotoxicity. Inorg. Chim. Acta.

[81] Anderson H.L., Anderson S., Sanders J.K.M. (1995). Ligand binding by butadiyne-linked porphyrin dimers, trimers and tetramers. J. Chem. Soc. Perkin Trans. 1.

