# Supplementary material for: Synthesis of Terpyridines: Simple Reactions—What Could Possibly Go Wrong?
Source: Molecules. 2019 May 9;24(9):1799. doi: 10.3390/molecules24091799 (PMC6539753; doi:10.3390/molecules24091799)
Supplement: Supplementary file 1 [file molecules-24-01799-s001.pdf]

Supporting information

Synthesis of Terpyridines: Simple Reactions – What could Possibly Go Wrong?

Dalila Rocco, Catherine E. Housecroft and Edwin C. Constable *

University of Basel, Department of Chemistry, BPR 1096, Mattenstrasse 24a, CH-4058 Basel, Switzerland; dalila.rocco@unibas.ch; catherine.housecroft@unibas.ch; alessandro.prescimone@unibas.ch

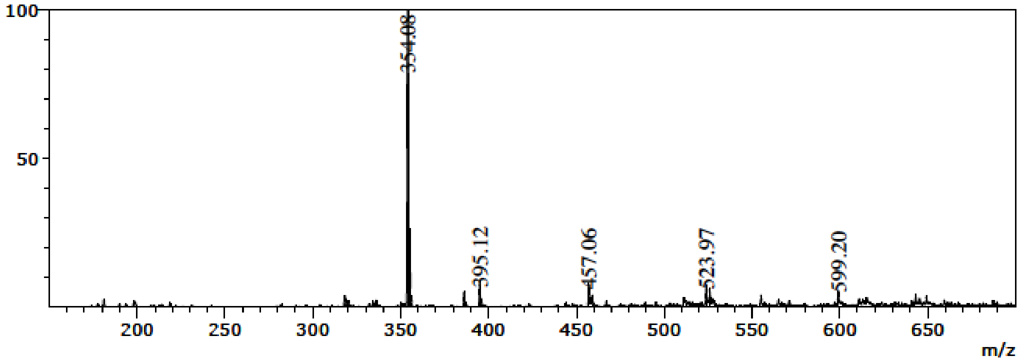

Figure S1. Electrospray mass spectrum of **5a**.

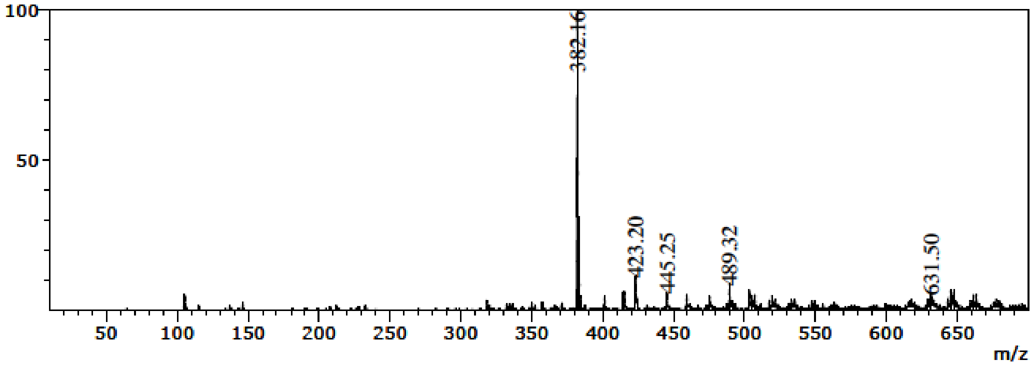

Figure S2. Electrospray mass spectrum of **5c**.

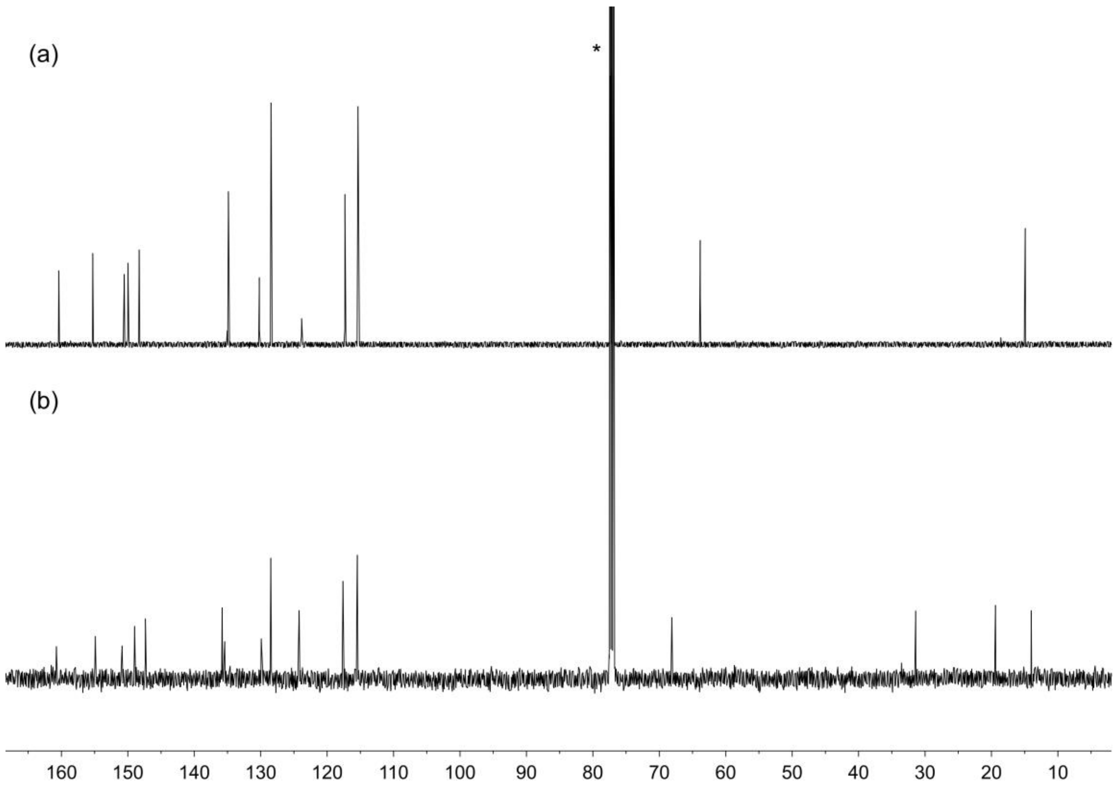

Fig. S3. $^{13}C\{^{1}H\}$ NMR (126 MHz, $CDCl_3$, 298 K) spectra of (a) **5a** and (b) **5c**. * = $CDCl_3$.

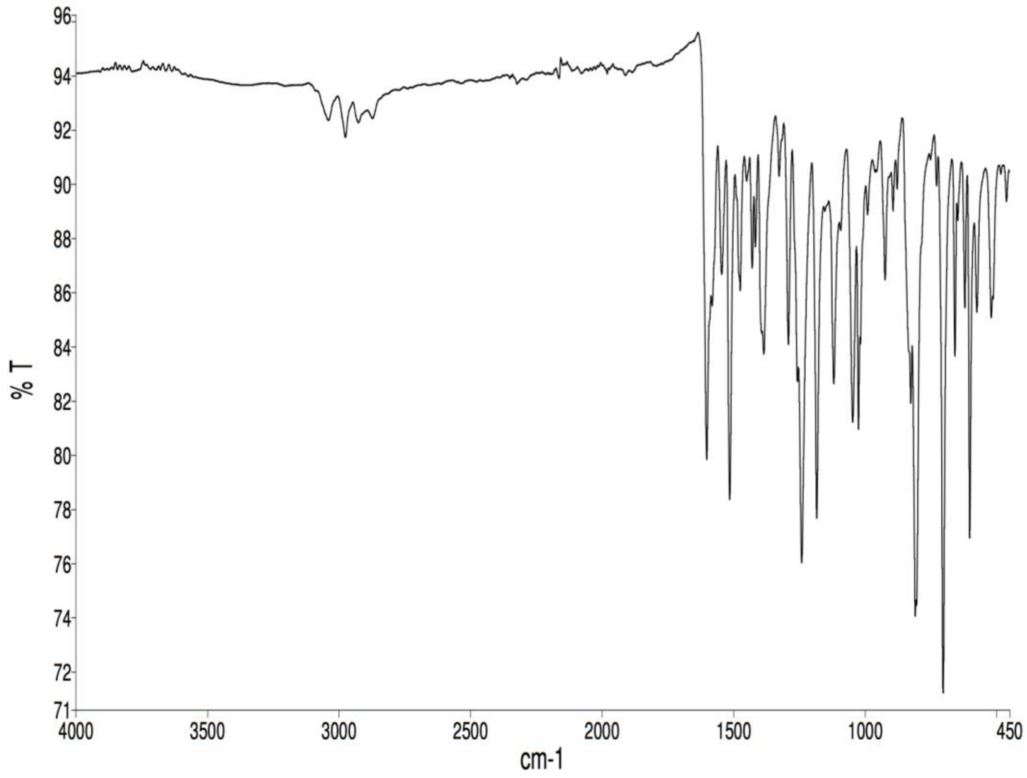

Fig. S4. IR spectrum of solid **5a**.

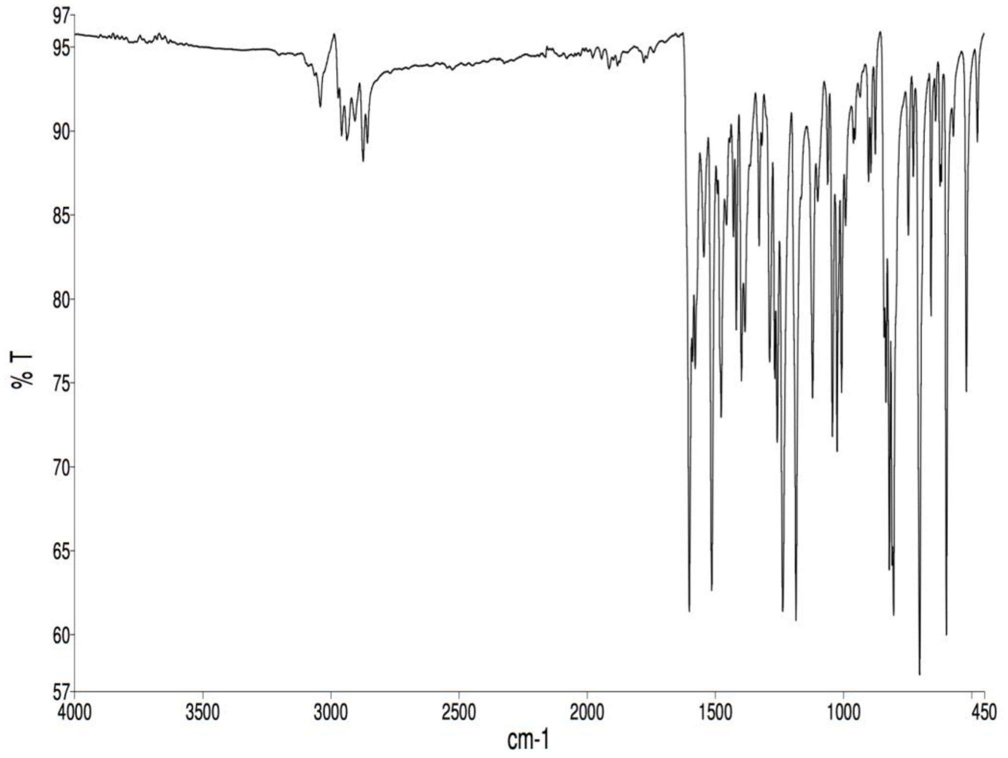

Fig. S5. IR spectrum of solid **5c**.

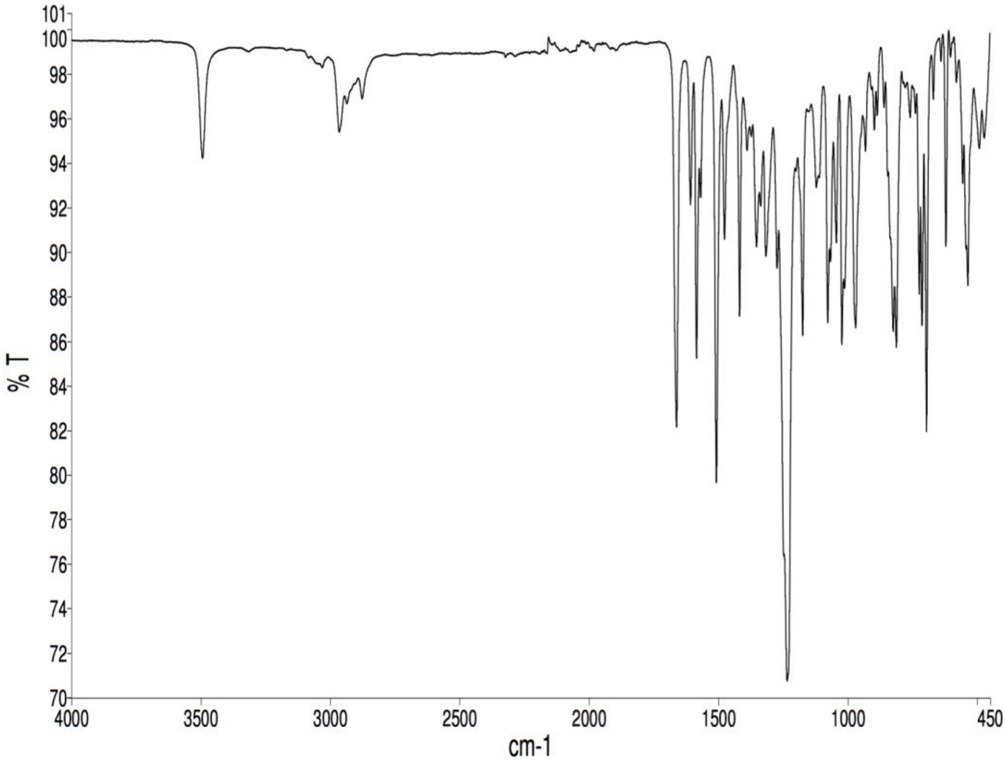

Fig. S6. IR spectrum of solid **6b**.

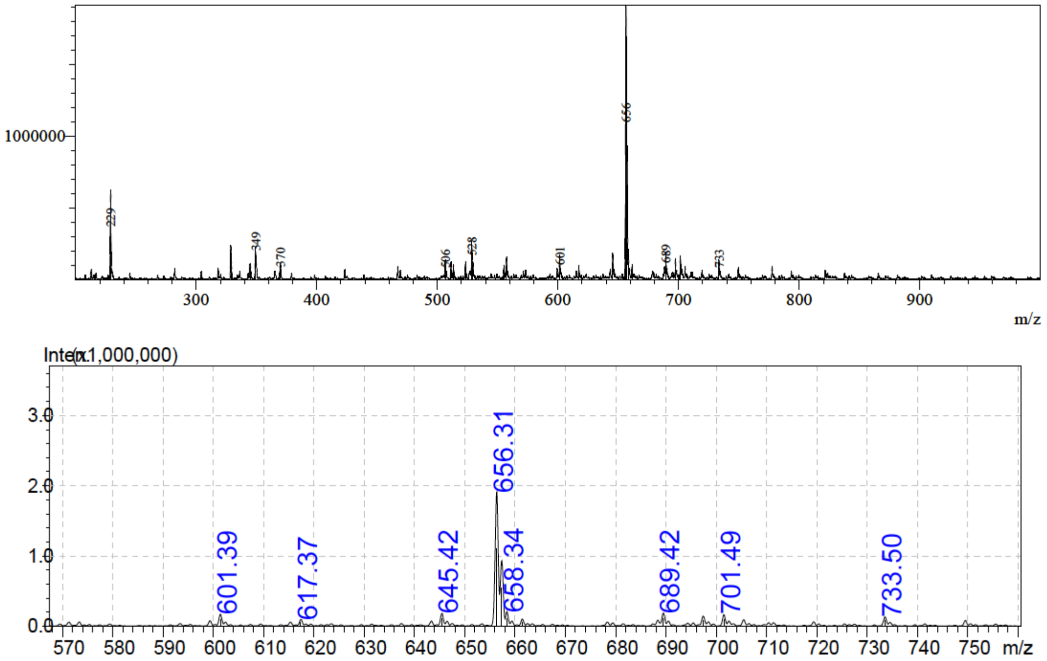

Figure S7. Electrospray mass spectrum of **6b**.

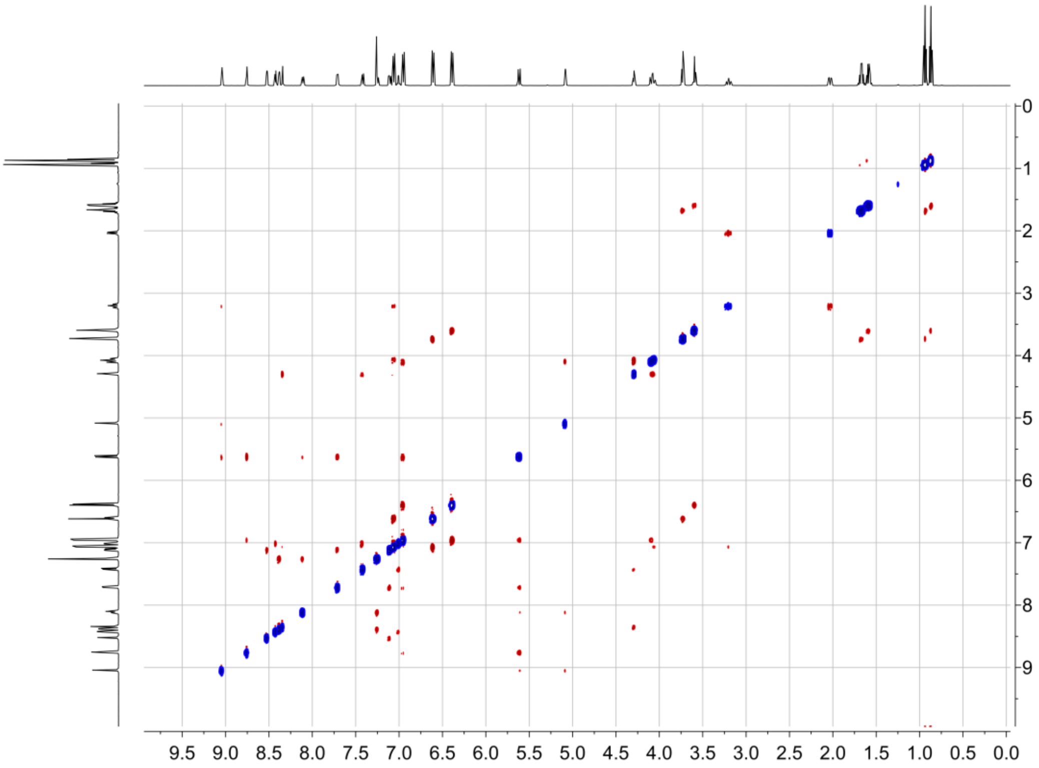

Figure S8. NOESY spectrum of **6b** (500 MHz, $CDCl_3$, 298 K).

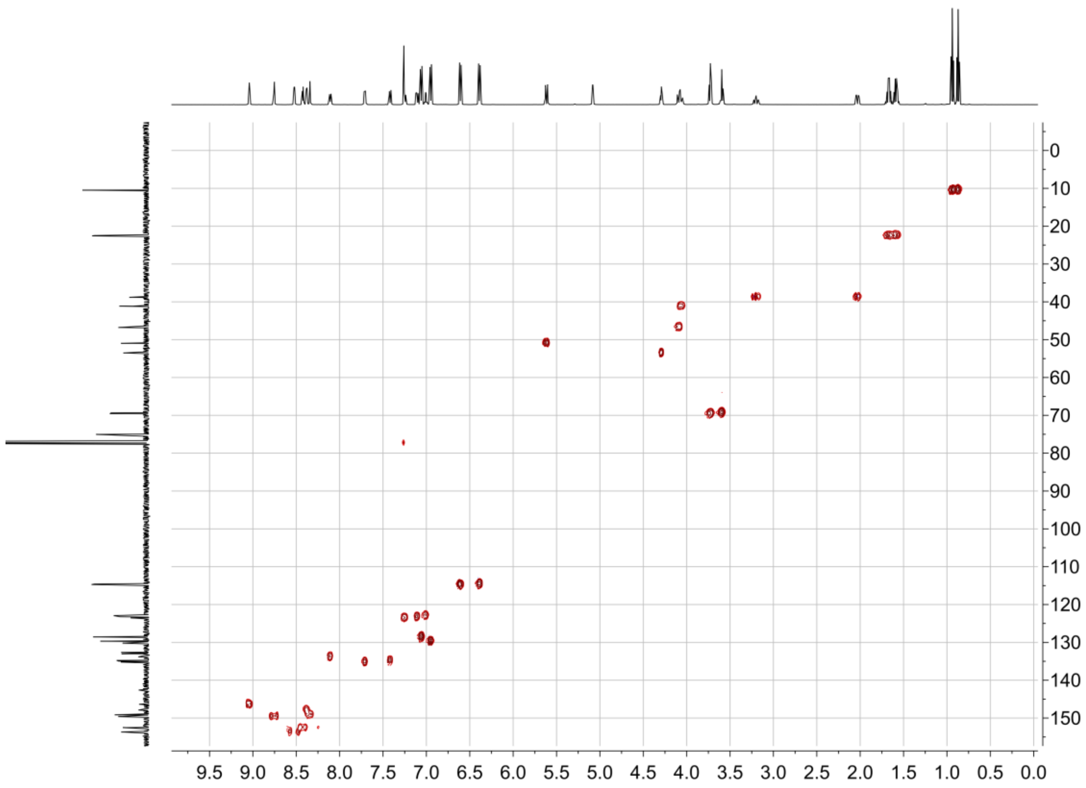

Figure S9. HMQC spectrum of **6b** (500 MHz, $CDCl_3$, 298 K).

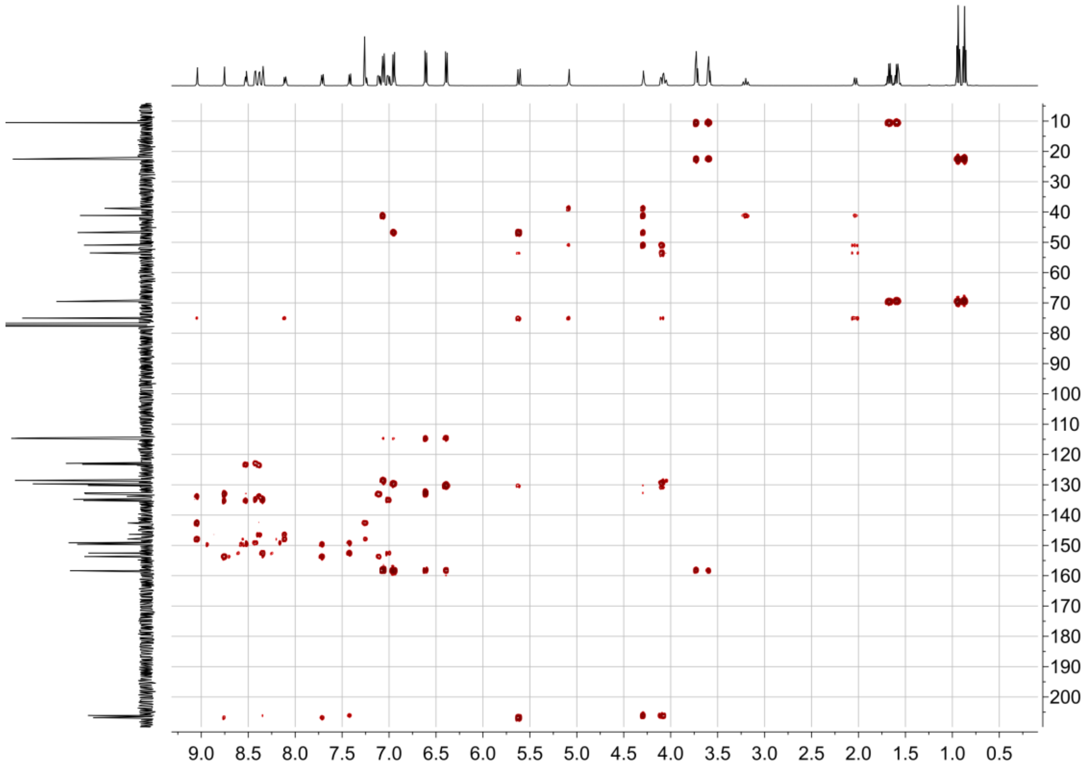

Figure S10. HMBC spectrum of **6b** (500 MHz, $CDCl_3$, 298 K).

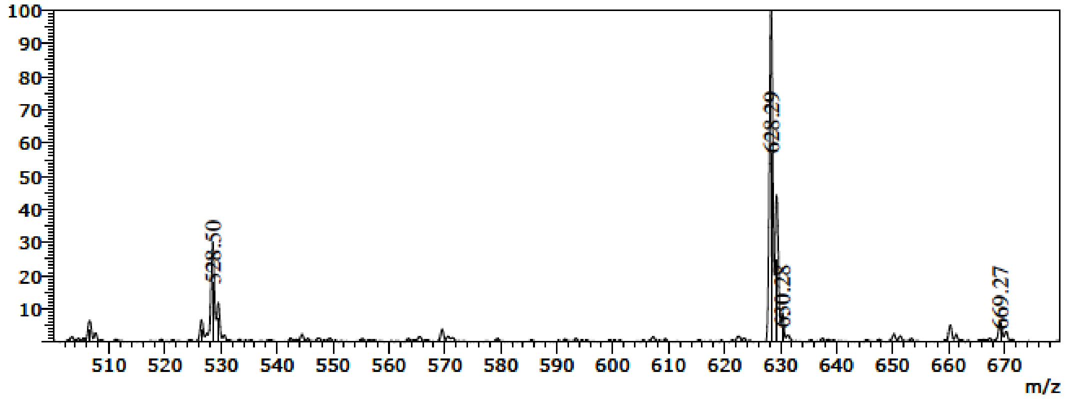

Figure S11. Electrospray mass spectrum of **6a**.

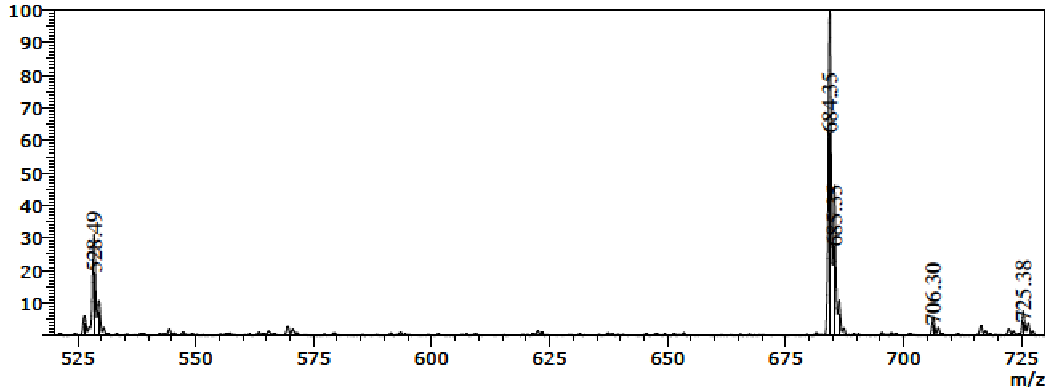

Figure S12. Electrospray mass spectrum of **6c**.

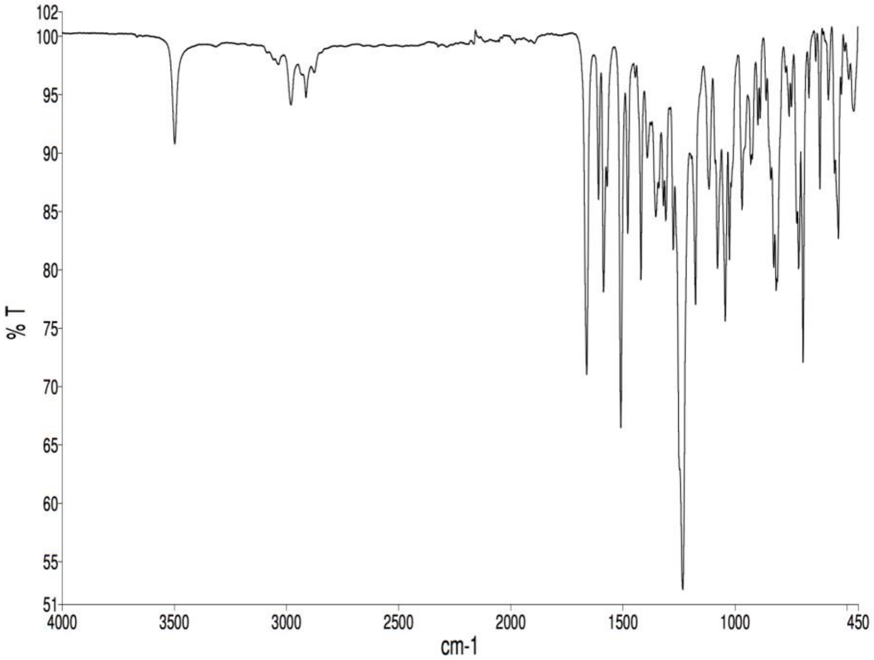

Figure S13. IR spectrum of solid **6a**.

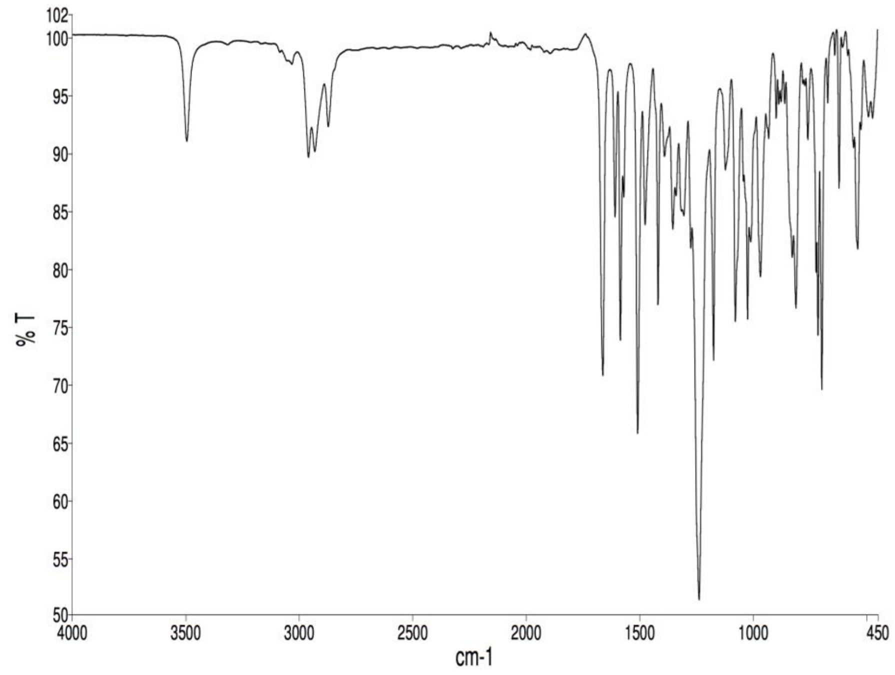

Figure S14. IR spectrum of solid **6c**.
